# Associations of incompetent lip seal and mouth breathing with stress in Japanese young adults: roles of oral function and nasal health

**DOI:** 10.1186/s12903-026-08030-1

**Published:** 2026-03-06

**Authors:** Yasutaka Kaihara, Mitsue Nagamine, Ryunosuke Hasegawa, Emi Inada, Yukiko Nogami, Yuki Kiyokawa, Daisuke Murakami, Issei Saitoh

**Affiliations:** 1 Department of Pediatric Dentistry, School of Dentistry, Osaka Dental University, 5-17 Otemae 1 choume, Chuo-ku, Osaka-shi, 540-0008 Japan; 2 Institute for Liberal Arts, Institute of Science Tokyo, Tokyo, 152-8550 Japan; 3 School of Environment and Society, Department of Social and Human Sciences, Institute of Science Tokyo, Tokyo, 152-8550 Japan; 4 Department of Pediatric Dentistry, Kagoshima University Graduate School of Medical and Dental Sciences, Kagoshima, 890-8544 Kagoshima Japan; 5 Takarazuka University of Medical and Health Care, Takarazuka, 665-0006 Hyogo Japan; 6 Department of Pediatric dentistry, Division of Oral Structure, Function and Development, Asahi University, School of Dentistry, Mizuho, Gifu 501-0296 Japan; 7 Keyakinomori Pediatric and Orthodontic Dental Clinic, Miyakonojo, Miyazaki, 885-0013 Japan

**Keywords:** Incompetent lip seal, Mouth breathing, Stress, Young adults, Oral function, Nasal disease, Structural equation modeling, Machine learning, AI (artificial intelligence)

## Abstract

**Background:**

Mouth breathing (MB) and incompetent lip seal (ILS) are common oral dysfunctions with potential repercussions for dentofacial development, systemic health, and psychological well-being. Although related, MB and ILS represent distinct conditions: ILS denotes difficulty maintaining lip closure at rest, whereas MB concerns the route of respiration. Despite growing recognition of their physical impacts, few studies have examined how MB and ILS are related to psychological stress in young adults.

**Methods:**

A cross-sectional study was conducted among 567 Japanese university students aged 18–27 years using a self-administered questionnaire. The survey included 53 items on oral conditions, lifestyle habits, and nasal symptoms, and 29 items from Section B of the Brief Job Stress Questionnaire to assess psychological stress. Exploratory factor analysis (EFA) was used to identify latent constructs. Extreme Gradient Boosting (XGBoost) was applied to classify high-stress individuals and highlight important predictors, while direct and indirect associations among ILS, MB, nasal disease, and stress were examined using structural equation modeling (SEM).

**Results:**

EFA extracted five latent factors: nasal disease, ILS and MB, oral hygiene and bad breath, condition of the teeth and gums, and lip dryness. XGBoost achieved moderate predictive performance (accuracy = 0.69, F1 = 0.74), with top predictors corresponding to ILS- and MB-related items (“Do you sleep with your mouth open?” and “Is your mouth often open during the day?”). SEM indicated that nasal disease had a direct association with stress, whereas ILS and MB indirectly influenced stress through oral symptoms such as lip dryness and bad breath. Lip dryness also showed a direct path to stress, suggesting its dual role as both a symptom and a potential stressor.

**Conclusion:**

ILS and MB are associated with higher stress levels in young adults primarily through indirect pathways involving oral discomfort. Lip dryness may mediate this relationship. Their partial independence from nasal disease supports the view that ILS and MB are distinct but interrelated conditions. These findings highlight the need for early screening and integrated management of oral and nasal function to promote psychological well-being.

**Supplementary Information:**

The online version contains supplementary material available at https://doi.org/10.1186/s12903-026-08030-1.

## Introduction and background

In recent years, increasing attention has been paid to the effects of mouth breathing (MB) and an incompetent lip seal (ILS) on oral function and overall health [1–15]. MB and ILS are common oral dysfunctions that can adversely affect dentofacial development and overall well-being. In this study, MB is defined as habitual airflow predominantly through the mouth at rest or during sleep and ILS as the inability to maintain a relaxed, closed-lip posture at rest without perioral muscle strain. Although related, MB and ILS are distinct: ILS refers to resting lip competence, whereas MB is related to the route of breathing. They often co-occur but are not necessarily present simultaneously. Clarifying these constructs is essential for accurately interpreting their health associations.

Previous studies have shown that MB and ILS can negatively affect various oral health domains, including malocclusion [1, 5], periodontal disease [6], and dental caries [7], as well as systemic outcomes such as sleep-disordered breathing and poor sleep quality [8, 9], aggravated allergic symptoms [10], and impaired physical development [11, 12]. Studies from the 1990s and 2000s first identified the local oral consequences of MB, such as gingival inflammation and open-bite malocclusion [4, 6]. Subsequent investigations expanded this view to include systemic and developmental effects in children [1, 5]. More recent systematic reviews have synthesized these findings, highlighting that mouth breathing and an incompetent lip seal can influence not only craniofacial morphology but also psychosocial and behavioral outcomes [3, 16]. These developments illustrate the growing recognition that oral dysfunctions have multifactorial impacts extending beyond the oral cavity.

While traditional research into oral health has focused on physical and functional issues, such as mastication, speech, and pain [1, 2], the psychological and emotional consequences of oral dysfunction remain underexplored. In Japan, Developmental Insufficiency of Oral Function (DIOF) has recently gained formal recognition as a pediatric diagnostic framework encompassing screening, assessment, and management of oral dysfunctions—including ILS and MB—within clinical and public-health practice [15]. This context underscores growing attention to MB/ILS as targets for screening and early preventive care, and motivates studying their relevance beyond childhood into young adulthood. Our previous studies examined how ILS and MB affect children’s oral and systemic health, contributing to the formal recognition of DIOF and the development of assessment tools, such as lip-closure strength tests [11–14]. These investigations have laid the groundwork for exploring how oral dysfunction influences mental well-being and oral-health-related quality of life (OHRQoL) in later developmental stages.

However, compared with functional and morphological outcomes, fewer epidemiological studies have evaluated how ILS and MB relate to psychological stress, a key dimension of OHRQoL. Importantly, a recent meta-analysis demonstrated that chronic sleep loss can significantly impair emotional regulation and increase vulnerability to stress and mood disturbances [17]. Thomson et al. reported an association between emotionality and OHRQoL [18]. Recent literature has also highlighted a bidirectional relationship between stress and oral health—psychological distress may exacerbate oral symptoms (e.g., dryness, discomfort), while symptomatic oral states may, in turn, increase perceived stress [16, 19]. However, large-sample, quantitative examinations of whether MB and ILS are associated—directly or indirectly—with stress in young adults remain limited. Consequently, despite heightened attention to these issues due to the establishment of DIOF [15], existing research still lacks comprehensive analyses of how MB and ILS directly affect stress and OHRQoL in adults who have completed their growth phase.

This study aimed to clarify the structural relationships between ILS, MB, oral symptoms, nasal disease, and psychological stress in a sample of young adults. Exploratory factor analysis (EFA) was used to identify latent constructs, Extreme Gradient Boosting (XGBoost) to highlight features related to higher stress, and structural equation modeling (SEM) to test direct and indirect associations—particularly whether MB and ILS are related to stress via oral symptoms such as lip dryness and bad breath. Throughout, we avoid causal claims and interpret the paths as associations consistent with the cross-sectional design.

## Materials and methods

### Study design

This was a cross-sectional study investigating the associations among ILS, MB, nasal disease, and psychological stress in young Japanese adults.

### Participants and data collection

A total of 567 Japanese university students aged 18–27 years (302 male and 265 female) participated in the study. Participants were recruited through university mailing lists and online announcements at two institutions: a women’s junior college in Gifu Prefecture and a university in Tokyo. After excluding incomplete or inconsistent responses, 567 valid datasets were analyzed. Data were collected between 2020 and 2022, during the COVID-19 pandemic. All participants were therefore described as “healthy,” meaning that they did not report any chronic or acute illness and were attending regular university activities without medical restrictions. The survey was anonymous and self-administered, covering items on oral conditions, lifestyle habits, nasal symptoms, and stress. All participants completed an anonymous online questionnaire (Google Forms) after reading an electronic consent statement describing the study purpose, details about voluntary participation, and data handling procedures. Only those who provided informed consent could access the questionnaire.

The self-administered questionnaire consisted of 10 items on medical history and 43 items assessing oral health status, lifestyle habits, and nasal symptoms (53 items total). These items were adapted from previously published large-scale surveys of oral function in Japanese children and adolescents [11–14]—only the wording was modified from caregivers reporting on their child’s behaviors (e.g., “Is your child…?”) to participants reporting on themselves (e.g., “Are you…?” or “Do you…?”) while preserving the original content and intent of each question. By applying the same items to participants aged 18–27 years, we aimed to explore whether ILS and MB persist into young adulthood, thereby informing the potential need for early intervention. Although formal psychometric validation has not yet been conducted, content validity was supported by referencing prior research and through expert consensus within the study team. The complete English wording of all 53 items is provided in Table 1 and Supplementary File 1.

**Table 1:** Questionnaire items on systemic disease history, oral conditions, lifestyle habits, and nasal symptoms

Item no.	Question item
Medical history of systemic diseases	
Q1	Have you ever been diagnosed with asthma?
Q2	Have you ever been diagnosed with atopic dermatitis?
Q3	Have you ever been diagnosed with allergic rhinitis?
Q4	Have you ever been diagnosed with adenoid hypertrophy?
Q5	Have you ever been diagnosed with bronchitis?
Q6	Have you ever been diagnosed with otitis media?
Q7	Have you ever been diagnosed with hay fever?
Q8	Do you catch a cold more than twice a year?
Q9	Do you have a fever over 38 °C more than twice a year?
Q10	Do you get swollen tonsils more than twice a year?
Lifestyle habits and oral status	
Q11	Do you get tired easily?
Q12	Are you a good riser?
Q13	Are you good at exercising?
Q14	Are you a restless sleeper?
Q15	Do you have round shoulders?
Q16	Does your nose become stuffed easily during the day?
Q17	Does your nose become stuffed easily while sleeping?
Q18	Do you sneeze often?
Q19	Do you often have a runny nose?
Q20	Do you often have a nosebleed?
Q21	Do you often have a sore throat?
Q22	Do you often fail to listen?
Q23	Are you a habitual snorer?
Q24	Is your mouth often dry?
Q25	Do people tell you that you have bad breath in the morning?
Q26	Do people tell you that you have bad breath during day?
Q27	Is your mouth often open during the day?
Q28	Do you sleep with your mouth open?
Q29	Can you keep your mouth closed for about 1 min?
Q30	Do you have an overbite?
Q31	Do you have an underbite?
Q32	Do you have an anterior open bite?
Q33	Can you speak clearly?
Q34	Are your lips often chapped?
Q35	Are your lips thick?
Q36	Is your upper lip turned upward?
Q37	Are your teeth visible between your upper and lower lips?
Q38	Are your lips droopy?
Q39	Are your lips often cracked?
Q40	Are your gums often swollen?
Q41	Are your gums easily stained?
Q42	Are your teeth easily stained?
Q43	Do you often have canker sores?
Q44	Do you have tartar build-up?
Q45	Do your meals consist of small servings?
Q46	Do you prefer soft food?
Q47	Do you drink water during meals?
Q48	Do you eat fast?
Q49	Are you a picky eater?
Q50	Do you chew food well?
Q51	Are you a noisy eater?
Q52	Do you keep your mouth closed when you eat?
Q53	Do you have food left in your mouth for a long time?

This self-administered questionnaire included 10 items on the medical history of systemic diseases and 43 items on lifestyle habits, oral condition, and nasal symptoms

Items Q1–Q10 were rated on a two-point scale (yes/no), while Q11–Q53 were rated on a four-point scale (yes, maybe yes, maybe no, no)

Items related to medical history were recorded on a two-point scale (yes/no), whereas oral health, lifestyle, and nasal symptoms were rated on a four-point scale: yes, maybe yes, maybe no, and no). All participants provided informed consent before participation. The first page of the Google Form included an explanation of the study as well as an informed consent statement. The study was conducted in accordance with the Declaration of Helsinki. Participants who agreed were permitted to access and complete the questionnaires. This study was approved by the Ethics Review Committee of the Ogaki Women’s Junior College (approval no. R2-2) and the Institute of Science Tokyo (Approval No. 2024307).

### Stress assessment

Psychological and physical stress reactions were assessed using Section B of the English version of the new Brief Job Stress Questionnaire (BJSQ) developed by Japan’s Ministry of Health, Labour and Welfare [20, 21]. Section B comprises 29 items covering five psychological domains (vigor, anger/irritability, fatigue, anxiety, and depression) and one physical domain (somatic complaints) (Table 2).

**Table 2:** Items from Section B of the Brief Job Stress Questionnaire (BJSQ) and their subscales

	Question item	Scale
Q1	I have been very active	Vigor (reverse scored)
Q2	I have been full of energy	Vigor (reverse scored)
Q3	I have been lively	Vigor (reverse scored)
Q4	I have felt angry	Anger/irritability
Q5	I have been inwardly annoyed or aggravated	Anger/irritability
Q6	I have felt irritable	Anger/irritability
Q7	I have felt extremely tired	Fatigue
Q8	I have felt exhausted	Fatigue
Q9	I have felt weary or listless	Fatigue
Q10	I have felt tense	Anxiety
Q11	I have felt worried or insecure	Anxiety
Q12	I have felt restless	Anxiety
Q13	I have been depressed	Depression
Q14	I have thought that doing anything was a hassle	Depression
Q15	I have been unable to concentrate	Depression
Q16	I have felt gloomy	Depression
Q17	I have been unable to handle work	Depression
Q18	I have felt sad	Depression
Q19	I have felt dizzy	Physical Stress Reaction (Somatic complaints)
Q20	I have experienced joint pains	Physical Stress Reaction (Somatic complaints)
Q21	I have experienced headaches	Physical Stress Reaction (Somatic complaints)
Q22	I have had a stiff neck and/or shoulders	Physical Stress Reaction (Somatic complaints)
Q23	I have had lower back pain	Physical Stress Reaction (Somatic complaints)
Q24	I have had eyestrain	Physical Stress Reaction (Somatic complaints)
Q25	I have experienced heart palpitations or shortness of breath	Physical Stress Reaction (Somatic complaints)
Q26	I have experienced stomach and/or intestinal problems	Physical Stress Reaction (Somatic complaints)
Q27	I have lost my appetite	Physical Stress Reaction (Somatic complaints)
Q28	I have experienced diarrhea and/or constipation	Physical Stress Reaction (Somatic complaints)
Q29	I haven’t been able to sleep well	Physical Stress Reaction (Somatic complaints)

Items were categorized based on the original structure of the Brief Job Stress Questionnaire (BJSQ), following the official classification of stress responses recommended by the Ministry of Health, Labour and Welfare of Japan [16, 22]

Psychological Stress Response (Positive): Vigor (Items 1–3; reverse scored)

Psychological Stress Response (Negative): Anger/Irritability (Items 4–6), Fatigue (Items 7–9), Anxiety (Items 10–12), Depression (Items 13–18)

Physical Stress Response: Somatic complaints (Items 19–29)

This classification also aligns with common international frameworks for stress and mental health research (e.g., emotional reactions, fatigue, somatic complaints)

Vigor (Items 1–3) was reverse scored so that higher scores indicate greater stress, aligning the direction of scoring with the other negative stress response items

This procedure is consistent with the original scoring method of the BJSQ, in which high vigor represents low stress

Participants rated each item on a four-point Likert scale (1 = strongly disagree to 4 = strongly agree). The Vigor subscale (items 1–3) was reverse scored so that higher total scores uniformly reflected higher stress levels. This procedure aligns with the BJSQ’s original scoring guidelines, which define high vigor as low stress and therefore requires reversal when computing the total stress level.

The composite stress score was used as a continuous variable in SEM. For the machine learning analysis using XGBoost, the score was binarized at the median to classify the participants into high-stress (1) and low-stress (0) groups. This unified treatment of the total score is consistent with previous applications of the BJSQ and reflects its intended use as a comprehensive indicator of stress.

### Statistical analysis

#### Exploratory factor analysis

EFA was conducted on 43 questionnaire items related to oral symptoms, lifestyle habits, and nasal conditions to extract latent variables. The maximum likelihood method with a promax rotation was used. The number of factors was determined according to the Kaiser-Guttman criterion (eigenvalues > 1) and scree plot inspection. Items with loadings exceeding 0.40 were retained. Factor structures were used to inform the subsequent SEM. The sample size (*n* = 567) exceeded the recommended minimum for factor analysis (10 participants per item).

### Machine learning: XGBoost

To identify the predictors of high stress, an XGBoost classification model (XGBClassifier, random_state = 42) was developed. The input variables included all the questionnaire items. The dataset was randomly divided into training (80%) and testing (20%) subsets.

Hyperparameters, such as n_estimators, max_depth, and learning_rate, were optimized using a grid search with 3-fold cross-validation. The model performance was evaluated using precision, recall, F1-score, and feature importance. The results were reported for both high- and low-stress classes.

### Structural equation modeling

Based on the constructs identified by EFA and the key variables highlighted by XGBoost, an SEM was developed to evaluate the relationships between the latent variables (e.g., MB, ILS, nasal disease, and lip dryness) and psychological stress. The model included both direct and indirect paths. The model fit was evaluated using the following indices: Comparative Fit Index (CFI), Goodness of Fit Index (GFI), Adjusted Goodness of Fit Index (AGFI), Tucker-Lewis Index (TLI), Root Mean Square Error of Approximation (RMSEA), Akaike Information Criterion (AIC), and Bayesian Information Criterion (BIC). Statistical significance was set at *p* < 0.05.

Machine learning and SEM were performed using Python (version 3.12.6) in a Jupyter Notebook environment (version 7.3.2). The following libraries were used: pandas (version 2.1.1), numpy (version 1.25.2), scikit-learn (version 1.3.2), xgboost (version 3.0.0), and semopy (version 2.4.3). Differences were considered statistically significant in all analyses.

## Results

### Preliminary analysis: self-reported indicators of mouth breathing and incompetent lip seal

**Fig. 1:**
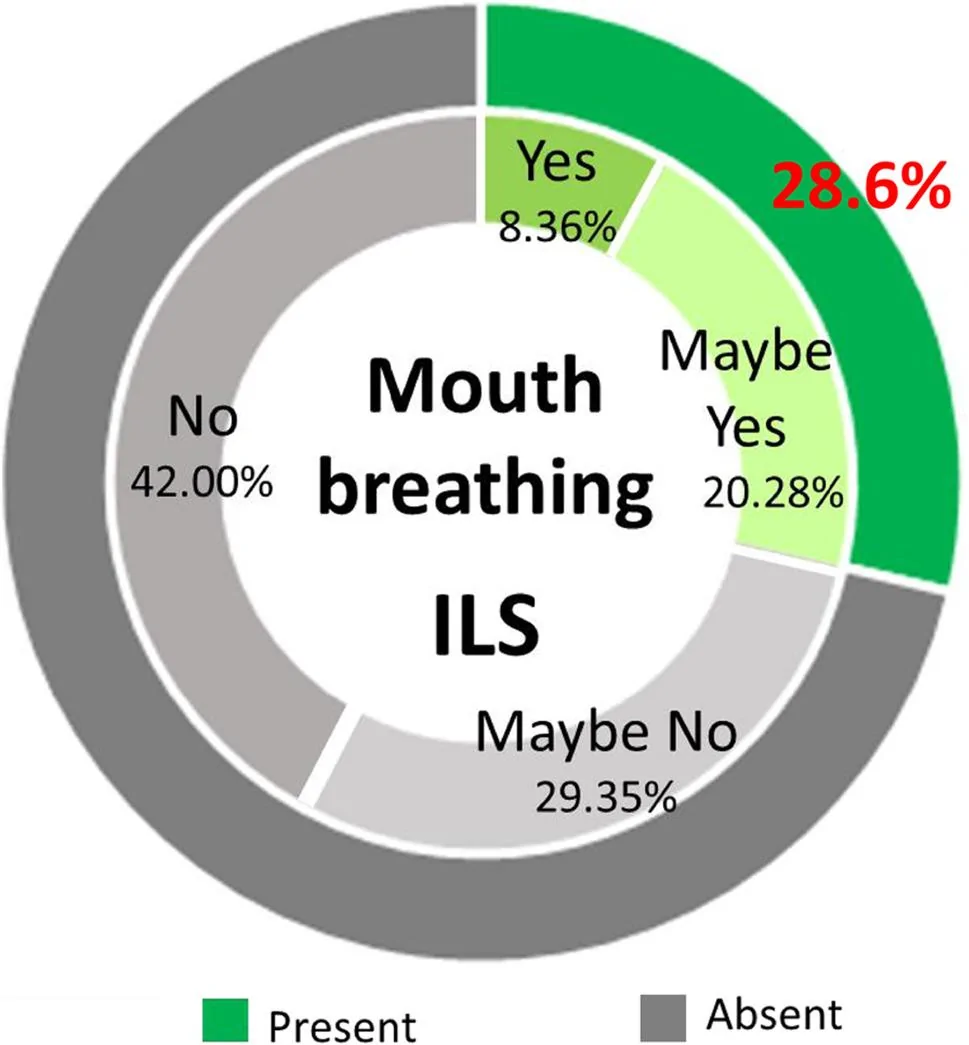
Self-reported frequency of mouth breathing and incompetent lip seal (ILS). Responses to the questionnaire item: “Is your mouth often open during the day?” representing participants' self-reports. Present indicates responses of “Yes” or “Maybe yes.”. Absent indicates responses of “No” or “Maybe no.”

The mean age of the participants was 20.4 years (SD = 1.8). In this study, following previous research using pediatric and adolescent populations [11–14], we interpreted Question 27 (“Is your mouth often open during the day?”) as a self-reported indicator of ILS and MB. This approach is consistent with earlier studies that used this item to screen for ILS tendencies in large-scale surveys. From responses of “Yes” or “Maybe Yes,” 28.6% of participants were classified as having signs of ILS or MB (Fig. 1).

### Exploratory factor analysis

EFA extracted five latent factors from the 43 items: (1) nasal disease, (2) ILS and MB, (3) oral hygiene and bad breath, (4) condition of teeth and gums, and (5) lip dryness. Consequently, 23 of the 43 items were retained and classified into these five factors (Table 3). The Kaiser–Meyer–Olkin (KMO) measure was 0.792, and Bartlett’s test of sphericity yielded *P* < 0.001, confirming the adequacy of the sample for factor analysis. The cumulative contribution ratio was 49.7%. Items with factor loadings ≥ 0.40 were used to compute mean factor scores for subsequent analyses. These latent constructs were entered into the SEM as observed indicators.

**Table 3:** Pattern matrix of the exploratory factor analysis

Item no.		Factor 1	Factor 2	Factor 3	Factor 4	Factor 5
	Question item	Disease of the nose	Incompetent lip seal Mouth breathing	Condition of teeth and gum	Bad breath	Lip Dryness
Q16	Does your nose become stuffed easily while sleeping?	0.805				
Q19	Do you often have a nosebleed?	0.790				
Q17	Do you sneeze often?	0.732				
Q18	Do you often have a runny nose?	0.674				
Q37	Are your teeth visible between your upper and lower lips?		0.808			
Q38	Are your lips droopy?		0.747			
Q30	Do you have an overbite?		0.579			
Q28	Do you sleep with your mouth open?		0.440			
Q27	Is your mouth often open during the day?		0.407			
Q42	Are your teeth easily stained?			0.646		
Q44	Do you have tartar build-up?			0.596		
Q40	Are your gums often swollen?			0.581		
Q41	Are your gums easily stained?			0.454		
Q26	Do people tell you that you have bad breath during day?				1.002	
Q25	Do people tell you that you have bad breath in the morning?				0.626	
Q34	Are your lips often chapped?					1.019
Q39	Are your lips often cracked?					0.419

Pattern matrix derived from exploratory factor analysis (EFA) using Promax rotation and maximum likelihood estimation

Items were grouped into five latent factors. Factor loadings > 0.4 were considered meaningful

Table 4 presents the factor correlation matrices. Factor 2 (ILS and MB) was the most strongly correlated with Factor 3 (*r* = 0.558), followed by Factors 5 (*r* = 0.349), 1 (*r* = 0.326), and 4 (*r* = 0.228). All correlation coefficients were statistically significant (*p* < 0.001).

**Table 4:** Factor correlation matrix

	Factor 1	Factor 2	Factor 3	Factor 4	Factor 5
	Disease of the nose	Incompetent lip seal/Mouth breathing	Condition of teeth and gums	Bad breath	Lip Dryness
Factor 1	1	0.326***	0.367***	0.177***	0.284***
Factor 2		1	0.558***	0.228***	0.349***
Factor 3			1	0.432***	0.410***
Factor 4				1	0.197***
Factor 5					1

***P < 0.001

Factor correlation matrix showing intercorrelations among the five latent constructs identified by EFA

All correlation coefficients are statistically significant (***p < 0.001)

### Artificial intelligence-based machine learning: XGBoost

The XGBoost model classified the participants into high- and low-stress groups with an overall accuracy of 0.69. For the high-stress group, the model achieved a precision of 0.67, recall of 0.82, and F1-score of 0.74. For the low-stress group, precision was 0.76, recall was 0.59, and the F1-score was 0.66. These results indicate good predictive performance, particularly for identifying individuals at a high risk of stress. The detailed classification metrics are listed in Table 5.

**Table 5:** Classification report of the XGBoost model

Class	Precision	Recall	F1-score	Support
0 (Low Stress)	0.76	0.59	0.66	70
1 (High Stress)	0.67	0.82	0.74	72

Performance metrics of the XGBoost classifier distinguishing high-stress and low-stress individuals

Precision, recall, F1-score, and support (sample size per class) are reported for each group

Among the top features contributing to the classification were “Is your mouth often open during the day?” (Q27), “Do you sleep with your mouth open?” (Q19), “Do you often have nasal congestion during sleep?” (Q7), and “Are your lips dry?” (Q40), all of which relate to ILS, MB, or associated nasal/oral symptoms.

These findings suggest that self-reported oral dryness, mouth opening, and poor sleep were common characteristics of students with higher stress levels. Although the predictive accuracy was moderate, it provides useful screening insight rather than clinical diagnostic precision, highlighting the potential for questionnaire-based identification of high-stress individuals.

### Structural equation modeling

**Fig. 2:**
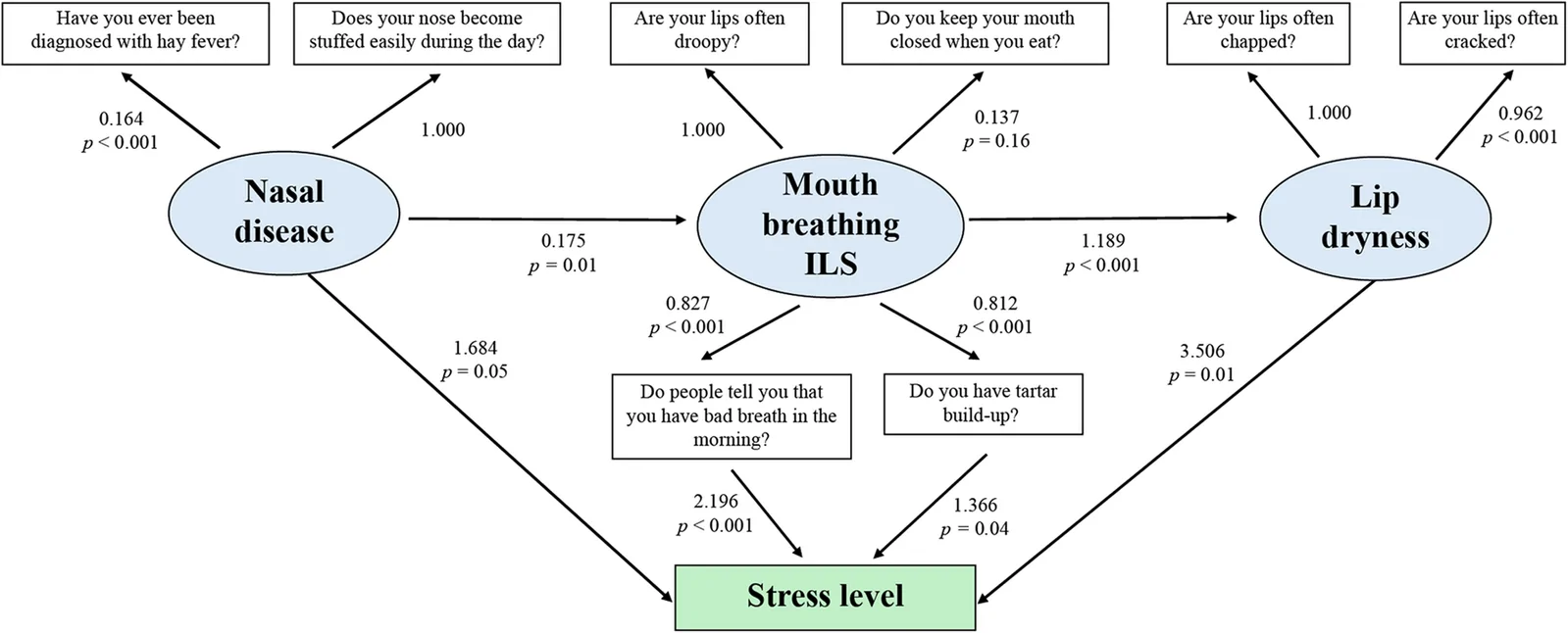
Path diagram of the final structural equation model. Values on the arrows represent unstandardized regression coefficients (B) with corresponding P-values. ILS = Incompetent Lip Seal. Model fit indices for this model are presented in Table 6

The final SEM model comprised three latent constructs—Nasal Disease (Q7, Q16), ILS/MB (Q25, Q37, Q44, Q52), and Lip Dryness (Q34, Q39)—and one observed variable (Stress level). The model showed an acceptable fit (Table 6). The final path diagram is shown in Fig. 2.

**Table 6:** Fit indices for the final structural equation model (SEM), indicating good model fit

Fit Index	Value
CFI (Comparative Fit Index)	0.952
GFI (Goodness of Fit Index)	0.915
AGFI (Adjusted Goodness of Fit Index)	0.86
NFI (Normed Fit Index)	0.915
TLI (Tucker-Lewis Index)	0.921
RMSEA (Root Mean Square Error of Approximation)	0.045
AIC (Akaike Information Criterion)	45.835
BIC (Bayesian Information Criterion)	145.663
LogLik (Log-Likelihood)	0.082

The following significant structural paths were identified:

- Nasal Disease → ILS/MB (B = 0.175, *p* = 0.01).
- ILS/MB → Lip Dryness (B = 1.189, *p* < 0.001).
- Lip Dryness → Stress Level (B = 3.506, *p* = 0.01).
- Nasal Disease → Stress Level (B = 1.684, *p* = 0.05).
- ILS/MB → Stress Level (B = 1.366, *p* = 0.04).

These results indicate that nasal disease exerts both direct and indirect effects on psychological stress.

The indirect pathway is mediated through ILS/MB and Lip Dryness, suggesting that oral dysfunctions may contribute to increased stress via both nasal and oral mechanisms. Notably, the item “Do you keep your mouth closed when you eat?” (Q52) showed a non-significant loading (*p* = 0.16), indicating a weak association with the ILS/MB construct in this model.

## Discussion

### Oral dysfunction and its link to stress

This study investigated the associations among ILS, MB, nasal disease, and psychological stress in young Japanese adults using a combined analytical approach of EFA, machine learning (XGBoost), and SEM. The SEM results revealed that nasal disease exerted both direct and indirect effects on psychological stress. Indirect effects were mediated through ILS/MB and lip dryness, indicating that oral dysfunctions may contribute to psychological burden via both nasal and oral pathways.

Although ILS/MB themselves were not directly linked to stress in the analyses, the structural model demonstrated that their impact operates primarily through oral symptoms such as lip dryness, tartar, and bad breath. These findings suggest that subjective oral discomfort serves as a key pathway connecting oral dysfunction to stress, consistent with previous studies reporting that oral dryness and nasal obstruction are associated with fatigue, irritability, and sleep disturbances [18, 19]. This highlights the importance of early recognition of oral symptoms even among healthy young adults. However, because of the cross-sectional nature of this study, causality cannot be inferred.

### Integration of EFA, XGBoost, and SEM

A key methodological strength of this study was the integration of traditional psychometric and modern data-driven approaches. EFA summarized questionnaire items into interpretable latent constructs representing oral, nasal, and lifestyle domains, clarifying the internal structure of the data. XGBoost, a machine learning model capable of detecting nonlinear and interaction effects, supported the validity of the EFA-derived constructs by identifying top predictors—such as lip dryness, mouth opening, and sleep quality—that corresponded closely to the “oral symptom” and “MB/ILS” factors.

Furthermore, XGBoost highlighted individual items (e.g., sleep quality, bad breath) that were not dominant in the factor solution, suggesting that machine learning can complement EFA by detecting item-specific and nonlinear associations with stress. SEM then integrated these findings into a unified model, demonstrating direct and indirect pathways from nasal disease to stress via ILS/MB and lip dryness.

Although the classification performance of XGBoost was moderate (accuracy = 0.69, F1 = 0.74), these values are acceptable for exploratory behavioral research and provide supplementary evidence supporting the robustness of the SEM findings. From a clinical standpoint, the model’s ability to identify key behavioral indicators—such as habitual mouth opening and oral dryness—is considered meaningful. These behavioral indicators are observable in routine dental or pediatric examinations and may provide practical cues for early detection and intervention in stress-related oral dysfunction among young adults. This three-step approach—EFA for structure, XGBoost for feature relevance, and SEM for relational validation—provides a comprehensive framework applicable to other psychosomatic domains in dental and behavioral research.

### Mediating role of oral symptoms

The mediating role of oral symptoms, particularly lip dryness, deserves special consideration. Lip dryness is known to reflect physiological mechanisms such as dehydration, increased oral airflow, and mucosal inflammation caused by habitual mouth opening [4, 7, 10]. These symptoms may increase self-perceived discomfort, thereby contributing to stress perception. Previous studies have also shown that psychological stress can itself exacerbate oral dryness, possibly through sympathetic activation or salivary-gland suppression [23, 24]. This bidirectional relationship may explain why oral symptoms are both consequences and mediators of stress. The indirect pathway observed in this study—Nasal Disease → ILS/MB → Lip Dryness → Stress—therefore highlights the importance of maintaining proper lip seal and nasal breathing to prevent secondary psychological strain. Our results are consistent with reports in children showing that MB and ILS are associated with emotional and behavioral problems [12–14, 25], suggesting that oral function and mental state are closely intertwined throughout development.

### Comparison with previous research in children

Previous pediatric studies have demonstrated that MB and ILS are associated with oral dysfunctions such as malocclusion, gingival inflammation, and reduced salivary flow [5, 6, 13, 14]. Saitoh et al. [13] reported that children with habitual mouth opening had higher rates of oral dryness and irritability, while Inada et al. [12] found that preschool children with ILS exhibited lower lip-closure force and reduced oral moisture. These studies focused mainly on morphological or developmental aspects during childhood. The present study extends this line of research to young adults, a population in which such investigations are limited.

Our results suggest that, despite the completion of major craniofacial growth, functional habits such as MB and ILS may persist as behavioral tendencies, manifesting as subjective oral symptoms rather than overt structural changes. This continuity supports the notion that ILS and MB represent long-term behavioral patterns rather than transient developmental immaturities.

Moreover, studies in adults have reported associations between oral dryness and heightened psychological stress or poor sleep quality [9, 23, 24]. For example, Fujimoto et al. [23] observed that university students with MB reported higher perceived stress and fatigue, consistent with our finding that oral dryness mediated the link between MB/ILS and stress.

Compared with earlier pediatric research that primarily addressed dental morphology or airway obstruction, the present study provides novel evidence for psychophysiological mechanisms underlying oral dysfunction in adulthood. Collectively, these comparisons indicate that, although the mechanical consequences of MB/ILS (e.g., malocclusion) diminish with age, their psychological consequences—oral discomfort and stress vulnerability—may persist. This highlights the need for longitudinal approaches spanning from childhood to young adulthood to fully understand the developmental trajectory of oral dysfunction and its psychosocial correlates.

Interestingly, the items “Do you keep your mouth closed when you eat?” (Q52) and “Are you a noisy eater?” (Q51) showed non-significant loadings in both the factor analysis and SEM in the present study, and neither was associated with stress. In contrast, previous surveys in preschool and primary school children extracted “not closing the mouth while eating” as a key factor reflecting oral dysfunction [12, 13]. From a functional perspective, failure to close the mouth while eating should affect chewing efficiency and swallowing, often accompanied by noisy eating [12, 13]. The discrepancy between children and young adults may be explained by the difference in respondents—parents for children versus self-reports for adults. While parents are likely to notice such behaviors because they are visually apparent and socially concerning, young adults may not recognize them as functional issues. This difference suggests that behaviors that parents find worrisome in children may go unnoticed by individuals themselves in adulthood. Therefore, early parental recognition and intervention during childhood are essential for preventing persistent mouth-opening and noisy-eating habits into adulthood. Improvements in ILS and MB are generally achieved through orthodontic or behavioral interventions rather than occurring spontaneously [25]. This emphasizes the need for proactive management to prevent the persistence of these oral dysfunctions and their psychological impacts.

### Methodological considerations and limitations

This study had several methodological advantages, including a large sample size (*n* = 567) suitable for both EFA and SEM, and the combined use of psychometric and data-driven analyses. However, several limitations should be acknowledged. First, because this was a cross-sectional study, causal relationships cannot be inferred. Second, all data were self-reported, and subjective judgments (e.g., self-assessment of ILS or MB) may not fully correspond to objective clinical measures. This self-report bias may have introduced misclassification errors, potentially attenuating true associations between oral dysfunction and stress. Third, the survey was conducted between 2020 and 2022 during the COVID-19 pandemic, and participants’ stress levels or oral behaviors may have been affected by social restrictions and prolonged mask wearing. Fourth, participants were recruited from two universities located in different regions (Tokyo and Gifu Prefecture); although many students lived independently in dormitories or apartments, regional and lifestyle differences may still have influenced stress perception or oral habits. Finally, the questionnaire was adapted from instruments validated in children [11–14] and has not yet undergone psychometric validation for young adults; confirmatory factor analysis and longitudinal reliability testing are warranted in future research.

Moreover, although the machine learning model demonstrated good classification performance, the lack of external validation using independent samples limited the robustness of the results. Future studies should incorporate external datasets to verify the predictive utility of this model. Furthermore, as the participants were university students, they may represent a socioeconomically advantaged segment of the young adult population, which could limit the generalizability of the findings to other demographic groups.

In addition, because the questionnaire relied on self-reported awareness, certain habitual or unconscious behaviors—such as resting mouth opening—may not have been fully captured. Unlike pediatric studies that commonly use caregiver- or clinician-reported observable behaviors, subjective self-assessment in young adults may lead to imprecision in measurement. This limitation could have attenuated factor loadings in the exploratory factor analysis and resulted in relatively conservative effect sizes in the structural equation model. Therefore, the observed associations should be interpreted with caution, and may not be generalizable to other populations. Future studies in young adults could complement subjective self-assessment with assessments of observable behaviors by healthcare providers. Comparison of subjective and objective assessments could then help confirm the validity of the scale.

### Strengths

Despite these limitations, this study had several notable strengths. It is one of the few investigations to examine the relationships among ILS, MB, and psychological stress in young adults. By integrating EFA, SEM, and machine learning (XGBoost), this study provides a multidimensional understanding of how oral and nasal factors are linked to stress. This mixed-method design enhances both the credibility and interpretability of the findings. Moreover, the inclusion of a relatively large and demographically diverse sample improves the generalizability of the results across different student populations.

### Implications for practice and research

These findings highlight the potential long-term effects of early oral dysfunctions on mental health. Recognizing the signs of ILS and MB during childhood may allow for earlier behavioral and clinical interventions, potentially mitigating psychological consequences later in life. This underscores the importance of interdisciplinary collaboration among dentists, pediatricians, and otolaryngologists to promote comprehensive oral–nasal health from a young age. Furthermore, longitudinal and interventional research is needed to examine whether improving oral function and breathing habits can contribute to better psychological well-being across the lifespan.

## Conclusion

This study demonstrated that ILS and MB in young adults are significantly associated with higher psychological stress. This association occurs primarily through indirect pathways involving oral symptoms such as bad breath, tartar accumulation, and lip dryness. Nasal disease was independently associated with stress, whereas MB and ILS showed a strong direct path to lip dryness regardless of nasal obstruction. This reinforced the need to address these conditions as distinct clinical entities. These findings, confirmed through SEM with a good model fit, highlight the importance of early screening and comprehensive intervention strategies targeting both oral and nasal health. Preventive efforts during childhood may be particularly effective and may benefit from interdisciplinary collaboration among dentists, pediatricians, and otolaryngologists. Although this cross-sectional study did not allow for causal inferences, it offered valuable insights into the mechanisms linking oral dysfunction and mental health. Future longitudinal and interventional studies are warranted to explore these pathways over time and evaluate the effectiveness of early behavioral or clinical interventions.

## Supplementary Information

Supplementary Material 1.

## Data Availability

The datasets generated and/or analyzed during the current study are not publicly available because of ethical restrictions, but are available from the corresponding author on reasonable request. Aggregated summary tables (i.e., the descriptive and analytic tables presented in the main manuscript) and the full questionnaire are included in this published article and its supplementary information files.

## Abbreviations

ILS: Incompetent Lip Seal
MB: Mouth breathing
BJSQ: Brief Job Stress Questionnaire
SEM: Structural equation model
DIOF: Developmental Insufficiency of Oral Function
OHRQoL: Oral-health-related quality of life
EFA: Exploratory Factor Analysis
CFI: Comparative Fit Index
GFI: Goodness of Fit Index
AGFI: Adjusted Goodness of Fit Index
TLI: Tucker-Lewis Index
RMSEA: Root Mean Square Error of Approximation
AIC: Akaike Information Criterion
BIC: Bayesian Information Criterion
