# Correction: Associations of incompetent lip seal and mouth breathing with stress in Japanese young adults: roles of oral function and nasal health

**DOI:** 10.1186/s12903-026-09273-8

**Published:** 2026-07-23

**Authors:** Yasutaka Kaihara, Mitsue Nagamine, Ryunosuke Hasegawa, Emi Inada, Yukiko Nogami, Yuki Kiyokawa, Daisuke Murakami, Issei Saitoh

**Affiliations:** 1 Department of Pediatric Dentistry, School of Dentistry, Osaka Dental University, 5-17 Otemae 1 choume, Chuo-ku, Osaka-shi, 540-0008 Japan; 2 Institute for Liberal Arts, Institute of Science Tokyo, Tokyo, 152-8550 Japan; 3 Department of Social and Human Sciences, School of Environment and Society, Institute of Science Tokyo, Tokyo, 152-8550 Japan; 4 Department of Pediatric Dentistry, Kagoshima University Graduate School of Medical and Dental Sciences, Kagoshima, Kagoshima 890-8544 Japan; 5 Takarazuka University of Medical and Health Care, Hyogo, Takarazuka, 665-0006 Japan; 6 Department of Pediatric dentistry, Division of Oral Structure, Function and Development, Asahi University, School of Dentistry, Mizuho, Gifu, 501-0296 Japan; 7 Keyakinomori Pediatric and Orthodontic Dental Clinic, Miyakonojo, Miyazaki, 885-0013 Japan

**Correction: BMC Oral Health 26, 661 (2026)**

**https://doi.org/10.1186/s12903-026-08030-1**

In this article [1], Table 4 includes the last row labeled “Factor 6” erroneously referring “six latent constructs” instead of five-factor structure. The corrected version of Table 4 is presented below to accurately reflect the intended five-factor model.

The original article has been corrected.

**Table 4:** Factor correlation matrix

	Factor 1	Factor 2	Factor 3	Factor 4	Factor 5
	Disease of the nose	Incompetent lip seal/Mouth breathing	Condition of teeth and gums	Bad breath	Lip Dryness
Factor 1	1	0.326 ***	0.367 ***	0.177 ***	0.284 ***
Factor 2		1	0.558 ***	0.228 ***	0.349 ***
Factor 3			1	0.432 ***	0.410 ***
Factor 4				1	0.197 ***
Factor 5					1

***P<0.001

Factor correlation matrix showing intercorrelations among the five latent constructs identified by EFA All correlation coefficients are statistically significant (***p<0.001)
